# Determining the Minimally Clinically Important Difference for the Disability Rating Scale in Persons With Chronic Traumatic Brain Injury

**DOI:** 10.1089/neur.2023.0038

**Published:** 2023-07-04

**Authors:** Flora M. Hammond, Jessica M. Ketchum, Vipul Vinod Patni, Bijan Nejadnik, Damien Bates, Alan H. Weintraub

**Affiliations:** ^1^ Department of Physical Medicine and Rehabilitation, Indiana University School of Medicine, Indianapolis, Indiana, USA.; ^2^ Traumatic Brain Injury Model Systems National Data and Statistical Center, Craig Hospital, Englewood, Colorado, USA.; ^3^ Talk Turkey Consulting, Shrirampur, India.; ^4^ SanBio, Inc., Mountain View, California, USA.; ^5^ Rocky Mountain Regional Brain Injury System and University of Colorado School of Medicine, Englewood, Colorado, USA.

**Keywords:** Disability Rating Scale, Extended Glasgow Outcome Scale, minimally clinically important difference, Spearman's correlations, traumatic brain injury

## Abstract

The Extended Glasgow Outcome Scale (GOSE) is accepted as the primary outcome measure in registrational studies for traumatic brain injury (TBI). The Disability Rating Scale (DRS) is used to assess functional progress from initial acute injury, through rehabilitation and reintegration into the community and life. For these reasons, the DRS is an alternative measure for assessing meaningful global outcomes in chronic TBI. The objective of this study was to determine the minimally clinically important difference (MCID) for the DRS in chronic TBI, by determining the magnitude of DRS change associated with the MCID for the GOSE of 1 point. This study is a retrospective analysis of the multi-center, prospective, longitudinal, Traumatic Brain Injury Model Systems National Database of persons with outcomes at 1, 2, and 5 years and every 5 years thereafter post-injury. Spearman's correlations for dynamic and static relationships between the DRS and GOSE were significant. For the 1-point MCID for the GOSE, the dynamic MCID estimate for the DRS of a −0.68-point change was calculated as the mean DRS change associated with the difference of the GOSE score between year 1 and year 2 (score range, 3–8), using all persons in the study (*n* = 11,102), whereas the exploratory static MCID estimate for the DRS of −1.28 points was calculated from the slope of the best-fit line between the DRS and GOSE at year 1 follow-up (score range, 3–8; *n* = 13,415). The final MCID for the DRS was calculated by using the triangulation method (i.e., the arithmetic mean of the dynamic and exploratory static MCID estimates), which resulted in a −1.0-point change. The significant correlation between the DRS and GOSE has allowed for the establishment of a −1.0-point MCID for the DRS, which supports the use of the DRS as an alternative primary outcome measure for chronic TBI research studies, including clinical trials.

## Introduction

The Extended Glasgow Outcome Scale (GOSE) is used to assess global outcomes in persons with brain injury, by categorizing global function using an 8-point ordinal scale that focuses on disability and functioning in daily life.^1^ The GOSE is accepted by the U.S. Food and Drug Administration (FDA) as a primary outcome measure in registrational studies for traumatic brain injury (TBI) and has been used in the majority of randomized controlled trials for acute TBI since 1980.^2,3^ In addition, the GOSE has been selected by the Common Data Elements project as its core outcome scale in TBI research, and the GOSE is recommended as an outcome scale by the National Institute of Neurological Disorders and Stroke and the National Institute of Child Health and Human Development.^4–6^ Although the GOSE is accepted as clinically relevant, and is the main global outcome scale for acute TBI, it is associated with limited reliability, ceiling effects, and dichotomization of outcomes and is insensitive to small but meaningful changes in chronic brain injury.^1,2^

The Disability Rating Scale (DRS) is used as the main alternative global outcome scale to the GOSE for TBI and was developed to assess the disability of persons with severe TBI, so that rehabilitative progress could be tracked from coma to return to the community.^1,7^ The DRS uses a 30-point ordinal scale that assesses eight functional areas which focus on neurological function and restrictions of daily living.^1,7^ A key difference between the DRS and GOSE is that the DRS places significant weight on the cognitive ability for self-care ratings of Feeding, Toileting, and Grooming, as well as ratings of Level of Functioning and Employability, which also assess physical function. In addition, the DRS is reported to be more sensitive than the Glasgow Outcome Scale in detecting improvement up to 2 years post-injury in persons with TBI who had undergone inpatient rehabilitation.^8^ Further, in a community-based study of persons with TBI who were between 2 and 9 years post-injury, the DRS Employability rating was able to detect a wide range of deficits, whereas most persons recorded maximum scores for the GOSE revealing a ceiling effect, which may limit the value of the GOSE in assessing long-term functional outcomes in chronic TBI.^9^

The minimally clinically important difference (MCID) was defined by Jaeschke and colleagues in 1989 as “the smallest difference in score in the domain of interest which patients perceive as beneficial and which would mandate, in the absence of troublesome side effects and excessive cost, a change in the patient's management.”^10^ It is noteworthy that for a given measurement scale in a clinical trial, comparison of the proportion of persons who achieved the MCID in the treatment and control groups may be more clinically meaningful than the statistical significance of the response between groups.^11^

The FDA has specified that a 1-point change of the GOSE is clinically meaningful for TBI clinical research and clinical management (SanBio, Inc., data on file, December 2019); therefore, in this study, we have defined the MCID for the GOSE as a 1-point improvement. Although there are no standard methods for determining the MCID, the MCID for the DRS in chronic TBI has been previously reported to be a: 1) −1-point change by the Delphi panel method^12^ and 2) −1.5-point change by triangulation of a Delphi panel estimate with distribution- and anchor-based estimates from a small randomized controlled clinical trial.^13^ However, in a large population of persons with chronic TBI, the MCID for the DRS has not been determined.

The Traumatic Brain Injury Model Systems (TBIMS) National Database (NDB) is a multi-center, prospective, longitudinal study with outcomes at 1, 2, and 5 years and every 5 years post-injury that was established to examine long-term outcomes after moderate-to-severe TBI.^14^ At the time of this analysis, the TBIMS NDB contained data on 14,083 persons at 1 year post-injury.^14^

This study is a retrospective analysis of the TBIMS NDB, in which the MCID for the DRS was determined by examining the relationships between the DRS and GOSE. The validity of this approach was confirmed by calculating Spearman's correlation coefficients for the dynamic and static relationships between the DRS and GOSE. The MCID for the DRS was determined by examining the dynamic and static relationships between the DRS and GOSE, based on the MCID for the GOSE of a 1-point change in the range of 3–8 points. The dynamic MCID estimate for the DRS included all persons who experienced change of both DRS and GOSE scores between year 1 and year 2, whereas the more conservative static MCID estimate required persons to have DRS and GOSE scores at year 1, which therefore may not have represented score changes over time. The final MCID for the DRS was calculated using the triangulation method of the arithmetic mean for the dynamic and static MCID estimates.^11,13^

This study is a retrospective analysis of the TBIMS NDB, which determined the MCID for the DRS that was associated with the MCID for the GOSE of a 1-point change in the range of 3–8 points and supported the DRS as an alternative primary outcome measure for chronic TBI interventional studies.

## Methods

### Participants

This study used the TBIMS NDB, a multi-center, prospective, longitudinal study that was established in 1987 to examine long-term outcomes after moderate-to-severe TBI.^14^ Follow-up visits in the TBIMS NDB are conducted at 1, 2, and 5 years post-injury and every 5 years thereafter up to 30 years post-injury.^14^ The TBIMS NDB inclusion criteria require persons to be in the acute stage of TBI and: 1) persons to have moderate-to-severe TBI defined by: i) post-traumatic amnesia for ≥24 h, ii) loss of consciousness for ≥30 min, iii) Glasgow Coma Scale (GCS) score of <13, or iv) trauma-related intracranial neuroimaging abnormalities; 2) admission to a TBIMS hospital emergency department within 72 h of injury; 3) persons to be aged ≥16 years of age at the time of injury; 4) persons to receive acute care and comprehensive inpatient rehabilitation at a TBIMS hospital; and 5) informed consent from the subject, guardian, or family member.^14^

The TBIMS NDB exclusion criteria are: 1) person's time of injury could not be determined within 12 h; 2) if, before admission to a comprehensive rehabilitation, a person left a designated TBIMS facility for >72 h; 3) the presence of lacerations or contusions of the face, eye, or scalp, without documented post-traumatic amnesia, loss of consciousness, or objective neurological findings that could be reasonably attributed to TBI; 4) the presence of fractures of the skull or facial bones without documented post-traumatic amnesia, loss of consciousness, or objective neurological findings that could be reasonably attributed to TBI; 5) the presence of primary anoxic, inflammatory, toxic, or metabolic encephalopathies that are not complications of TBI; and 6) the presence of ischemic or hemorrhagic stroke, airway obstruction, seizure disorders, intracranial surgery, or neoplasms.^15^ Deidentified data were obtained from the TBIMS National Data and Statistical Center and analyzed with the approval of the Indiana University Institutional Review Board.

This study used GOSE data, which was collected by the TBIMS NDB from July 1, 2000; therefore, persons in this study participated in the TBIMS NDB between July 1, 2000 and March 31, 2022. This study utilized follow-up data at 1, 2, and 5 years post-injury, with persons required to have both DRS and GOSE scores at only one of the time points in order to participate in the study (e.g., a person had both DRS and GOSE scores at year 1, but not necessarily at years 2 or 5). The requirement for both DRS and GOSE scores reduced the number of persons who were eligible to participate at each time point, such that follow-up data were available for 14,083 persons at year 1, 12,624 persons at year 2, and 10,310 persons at year 5. The characteristics of the study sample at year 1 and the TBIMS NDB population are summarized in Table 1 and show no notable differences between groups.

**Table 1.** Demographics

Characteristic	Study sample (year 1) (*n* = 14,083)	TBIMS NDB population (*n* = 19,044)
Male, %	73.0	73.7
White, %	68.6	65.9
Mean age, years	43.1	42.6
Education at injury, %		
Less than high school	22.1	24.4
High school/GED	34.8	35.1
Some college	23.9	22.2
At least a bachelor's degree	19.2	18.3
GCS on admission, %		
13–15 (mild)	43.5	42.4
9–12 (moderate)	14.6	15.4
3–8 (severe)	41.9	42.2
Mean/median GCS on admission	9.8/11.0	9.8/11.0
Mean/median duration of unconsciousness, days	7.7/2.0	7.6/2.0
Mean/median duration of post-traumatic amnesia, days	22/17	22/17
Primary cause of injury (%)	Vehicular (49.9)	Vehicular (49.2)

GED, Graduate Equivalency Diploma; GCS, Glasgow Coma Scale; TBIMS NDB, Traumatic Brain Injury Model Systems National Database.

It is noteworthy that at emergency department admission, 42.4% of the TBIMS NDB population had a GCS score of 13–15, indicating mild TBI (Table 1), while being classified as having moderate-to-severe TBI based on the TBIMS NDB inclusion criteria that uses multiple injury severity criteria as qualifying options, including GCS score, duration of post-traumatic amnesia, duration of loss of consciousness, and trauma-related intracranial neuroimaging abnormalities.

### Dynamic relationship between the Disability Rating Scale and Extended Glasgow Outcome Scale

The dynamic relationship between the DRS and GOSE was determined by plotting DRS change versus GOSE changes for the difference between year 1 and year 2. Spearman's correlation coefficient was calculated, with the null hypothesis that DRS and GOSE score changes were non-monotonic.

### Static relationship between the Disability Rating Scale and Extended Glasgow Outcome Scale

The static relationship between the DRS and GOSE (scale scores at specific time points post-injury) was determined by plotting DRS versus GOSE scores (GOSE range, 1–8) at 1, 2, and 5 years post-injury. Spearman's correlation coefficients were calculated for each time point, with the null hypothesis that DRS and GOSE scores were non-monotonic.

### Dynamic minimally clinically important difference for the Disability Rating Scale

The dynamic MCID for the DRS was determined for all persons who experienced a change of both DRS and GOSE scores between year 1 and year 2 and who had a GOSE score of 3–8 points at year 1, in which persons were separated into three groups: 1) persons who followed a consistent trend (*n* = 5730; i.e., improvement of both scales, worsening of both scales, or no change of both scales); 2) persons who were in soft defiance of the trend (*n* = 3064; i.e., improvement of DRS with no change of GOSE or worsening of DRS with no change of GOSE); and 3) persons who were in strong defiance of the trend (*n* = 2308; i.e., improvement of GOSE with worsening or no change of DRS or worsening of GOSE with improvement or no change of DRS; Table 2).

**Table 2.** Trends for DRS and GOSE Change

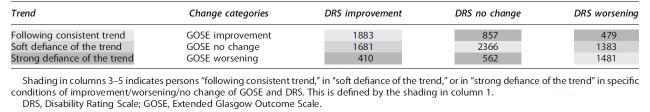

The dynamic MCID for the DRS was calculated for a GOSE change of 1 point on a scale of −7 to 5 for the difference between year 1 and year 2 for persons in all three trend groups (*n* = 11,102) and, as a subanalysis, persons who followed the consistent trend and were in soft defiance of the trend (*n* = 8794). Persons who were in strong defiance of the trend (*n* = 2308) were excluded from the subanalysis because they represented a divergent change on the DRS and GOSE scales. For the dynamic MCID for the DRS, descriptive statistics, including mean, median, and standard deviation (SD), were calculated.

### Static minimally clinically important difference for the Disability Rating Scale

The exploratory static MCID for the DRS required persons to have DRS and GOSE scores at year 1, which may not have represented change over time. The static MCID for the DRS was determined by plotting DRS scores versus GOSE score ranges of 3–8, 3–7, and 3–6 points at 1 year post-injury. Linear regression determined the slope of the best-fit line and coefficient of determination (*R*^2^) for each plot. For each GOSE score range, the slope of the best-fit line corresponded with the MCID for the DRS, which was associated with the MCID for the GOSE of a 1-point improvement.

### Final minimally clinically important difference for the Disability Rating Scale

There are no standard methods for determining the MCID; therefore, this study adopted an approach that used dynamic and exploratory static relationships between the DRS and GOSE to calculate the MCID for the DRS.^11^

Using the triangulation method, the final MCID for the DRS was calculated as: 1) the mean of the dynamic MCID (all three trend groups, *n* = 11,102) and the exploratory static MCID (GOSE score range of 3–8 points) that correlated with the MCID for the GOSE of a 1-point improvement; 2) as a subanalysis, the mean of the dynamic MCID (consistent trend and in soft defiance of the trend, *n* = 8794) and the exploratory static MCID (GOSE score range of 3–8 points), which correlated with the MCID for the GOSE of a 1-point improvement.

### Statistical analysis

Analyses were generated using Python (version 3.6.5; Anaconda, Inc., Austin, TX)/18.04.1-Ubuntu SMP System (8 cores 64GB RAM; Canonical Ltd, London, UK)/5.4.0-1043-Google Cloud Platform server (Google LLC, Mountain View, CA).

## Results

This study used data from persons with moderate-to-severe TBI who participated in the TBIMS NDB between July 2, 2000 and March 31, 2022, had follow-up data at 1 year (*n* = 14,083), 2 years (*n* = 12,624), and 5 years (*n* = 10,310) post-injury, and had both DRS and GOSE scores available for at least one of the time points.

### Dynamic relationship between the Disability Rating Scale and Extended Glasgow Outcome Scale

**FIG. 1.**
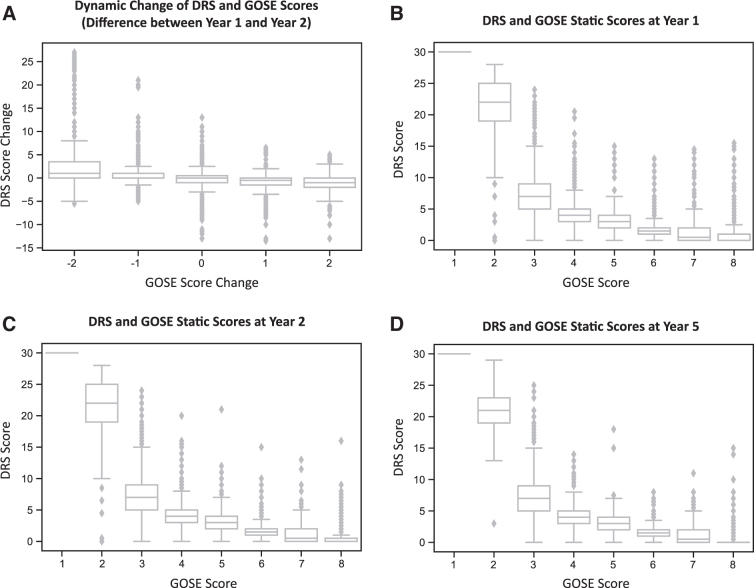
Spearman's correlation coefficients for the dynamic and static relationships between the DRS and GOSE. (**A**) Spearman's correlation coefficient of dynamic change for the DRS and GOSE (i.e., difference between year 1 and year 2) was −0.434 (95% CI: −0.445, −0.418; *p* < 0.01; *n* = 11,161). Spearman's correlation coefficients of static scores for the DRS and GOSE were: (**B**) −0.805 (95% CI: −0.812, −0.799; *p* < 0.01; *n* = 14,083) at year 1; (**C**) −0.809 (95% CI: −0.816, −0.802; *p* < 0.01; *n* = 12,634) at year 2; and (**D**) −0.835 (95% CI: −0.842, −0.828; *p* < 0.01; *n* = 10,130) at year 5. CI, confidence interval; DRS, Disability Rating Scale; GOSE, Extended Glasgow Outcome Scale.

Spearman's correlation coefficient of dynamic change for the difference between year 1 and year 2 for DRS and GOSE scores was moderate and statistically significant, showing a moderate monotonic relationship between scales in the study sample (−0.434 [95% CI: –0.445, −0.418], *p* < 0.01; difference between year 1 and year 2 [n = 11,161]; Fig. 1A).

### Static relationship between the Disability Rating Scale and Extended Glasgow Outcome Scale

Spearman's correlation coefficients for exploratory static scores for the DRS and GOSE at 1, 2, and 5 years after injury were high and statistically significant, showing a strong monotonic relationship between scales in the study sample (year 1: −0.805 [95% CI: −0.812, −0.799], *p* < 0.01 [*n* = 14,083]; year 2: −0.809 [95% CI: −0.816, −0.802], *p* < 0.01 [*n* = 12,634]; year 5: −0.835 [95% CI: −0.842, −0.828], *p* < 0.01 [n = 10,130]; Fig. 1B–D).

### Dynamic minimally clinically important difference for the Disability Rating Scale

Of the 11,102 persons who had both DRS and GOSE scores at both year 1 and year 2 (year 1 GOSE score range, 3–8) in this analysis, 5730 (51.6%) persons followed the consistent trend, 3064 (27.7%) persons were in soft defiance of the trend, and 2308 (20.7%) persons were in strong defiance of the trend (Table 2).

The dynamic MCID for the DRS was calculated using all three trend groups (*n* = 11,102; 100%), which resulted in a mean − 0.68-point change for the DRS (median, −0.50; SD, 1.65) that was associated with the MCID for the GOSE of a 1-point improvement between year 1 and year 2 (Table 3).

**Table 3.** Dynamic MCID for the DRS (*n* = 11,102)

GOSE change	DRS change			
	Mean	Median	SD	Count (*n*)
–7	28.30	29.0	2.20	53
–6	25.43	27.0	5.78	21
–5	13.71	6.0	12.07	78
–4	5.90	2.8	9.04	134
–3	7.30	2.5	10.44	258
–2	4.63	1.0	7.95	634
–1	0.57	0.0	1.84	1275
0	–0.13	0.0	1.75	5430
**1**	**–0.68**	**–0.5**	**1.65**	**1832**
2	–1.10	–1.0	1.69	886
3	–1.86	–2.0	2.43	296
4	–1.67	–2.0	2.06	165
5	–2.90	–2.0	2.95	40

Bold text indicates the MCID for the DRS associated with the MCID for the GOSE of a 1-point improvement in the overall study sample population (*n* = 11,102).

MCID, minimally clinically important difference; DRS, Disability Rating Scale; SD, standard deviation.

In a subanalysis, the dynamic MCID for the DRS was also calculated for persons who followed the consistent trend and were in soft defiance of the trend (*n* = 8794; 79.3%) and excluded persons who were in strong defiance of the trend (*n* = 2308; 20.7%). The subanalysis resulted in a mean − 1.80-point change for the DRS (median, −1.5; SD, 1.38), which was associated with the MCID for the GOSE of a 1-point improvement between year 1 and year 2 (Table 4).

**Table 4.** Dynamic MCID for the DRS (*n* = 8794)

GOSE change	DRS change			
	Mean	Median	SD	Count (*n*)
–7	28.30	29.0	2.20	53
–6	25.43	27.0	5.78	21
–5	14.76	7.0	11.75	73
–4	7.66	3.5	9.32	107
–3	9.81	3.5	10.63	199
–2	6.87	2.0	8.56	443
–1	1.96	1.5	1.69	585
0	–0.13	0.0	1.75	5430
**1**	**–1.80**	**–1.5**	**1.38**	**946**
2	–1.98	–2.0	1.38	570
3	–2.72	–2.5	2.02	225
4	–2.77	–3.0	1.42	111
5	–3.89	–4.0	2.59	31

Bold text indicates the MCID for the DRS associated with the MCID for the GOSE of a 1-point improvement in the population of persons who followed the consistent trend or were in soft defiance of the trend (*n* = 8794).

MCID, minimally clinically important difference; DRS, Disability Rating Scale; SD, standard deviation.

### Static minimally clinically important difference for the Disability Rating Scale

**FIG. 2.**
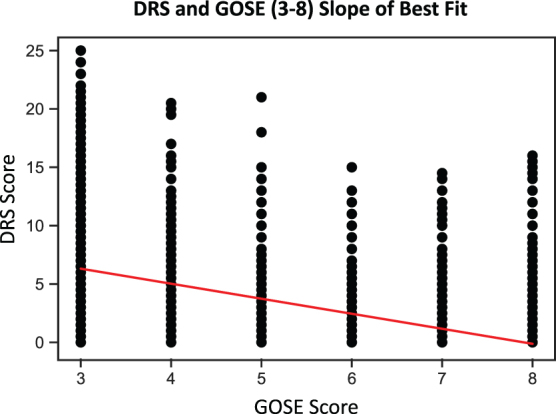
Static MCID for the DRS. The exploratory static MCID for the DRS was determined by plotting the DRS score versus the GOSE score range of 3–8 points at 1 year post-injury, which resulted in a slope of the best-fit line of −1.28 and coefficient of determination (*R*^2^) of 0.49. DRS, Disability Rating Scale; GOSE, Extended Glasgow Outcome Scale; MCID, minimally clinically important difference.

The exploratory static MCID for the DRS was calculated by plotting mean the DRS score versus GOSE score range of 3–8 points at 1 year post-injury (eliminating 4.6% of persons), which resulted in a slope of the best-fit line of −1.28 and a coefficient of determination (*R*^2^) of 0.49 (Fig. 2). The static MCID for DRS was therefore defined as −1.28 points over a GOSE score range of 3–8 points, which was associated with the MCID for the GOSE of a 1-point improvement.

Alternative analyses were conducted that plotted DRS scores versus GOSE score ranges of 3–7 (eliminating 25.3% of persons, of which 20.7% had a GOSE score of 8) and 3–6 (eliminating 38.2% of persons, of which 33.6% had GOSE scores of 7 and 8), which resulted in a slope of best-fit lines and *R*^2^ of −1.58 (*R*^2^ = 0.46) and −1.88 (*R*^2^ = 0.44), respectively (Supplementary Fig. S1A,B). These additional analyses resulted in exploratory static MCIDs for the DRS of −1.58 points (GOSE, 3–7) and −1.88 points (GOSE, 3–6), which were associated with the MCID for the GOSE of a 1-point improvement.

### Final minimally clinically important difference for the Disability Rating Scale

The final MCID for DRS of a −1.0-point change was calculated using the triangulation method as the mean of the dynamic MCID of a −0.68-point change (all three trend groups, *n* = 11,102) and the exploratory static MCID of a −1.28-point change (GOSE score range of 3–8 points; *n* = 13,415), which were associated with the MCID for GOSE of a 1-point improvement.

The subanalysis, which triangulated the dynamic MCID of a −1.80-point change (consistent trend and in soft defiance of the trend, *n* = 8794) and the exploratory static MCID of a −1.28-point change (GOSE score range of 3–8 points; *n* = 13,415) resulted in an MCID of −1.5 points, which was associated with the MCID for the GOSE of a 1-point improvement.

## Discussion

This study has established an MCID for the DRS of a −1.0-point change by correlating DRS change with the MCID for the GOSE of 1-point for patients with GOSE scores of 3–8 at year 1 in a retrospective analysis of the TBIMS NDB, a multi-center, prospective, longitudinal study.

### Dynamic and static relationships between the Disability Rating Scale and Extended Glasgow Outcome Scale

The dynamic correlation for the difference between year 1 and year 2 for DRS change and GOSE change was moderate (−0.434, *p* < 0.01), whereas strong static correlations were established between the DRS and GOSE scores of 1–8 at years 1, 2, and 5, which ranged from −0.805 to −0.835 (*p* < 0.01), indicating high concurrent validity between the two scales for up to 5 years post-injury. The strong static correlations reported in this study are supported by a previous study, in which a Spearman's correlation of −0.88 was reported for the relationship between the DRS and GOSE, also indicating high concurrent validity.^16^

The dynamic correlation was lower than static correlations, which was likely the result of measurement error, given that the dynamic analysis was based on two measurements (year 1 and year 2) in two scales for each person, whereas the static analysis was based on a single measurement (at years 1, 2, or 5) in two scales for each person. Therefore, four measurements were required per person for the dynamic correlation compared with two measurements per person for the static correlation, amplifying sources of variation and increasing measurement error.

Overall, the moderate-to-strong correlations between the DRS and GOSE in this large population of persons with chronic TBI supported using the relationship between the two scales to determine the MCID for the DRS based on the MCID for the GOSE of a 1-point change.

### Dynamic and static minimally clinically important difference estimates for the Disability Rating Scale

The analysis was led by the dynamic MCID estimate for the DRS, which correlated change of GOSE score with change of DRS score over time at the individual person level and therefore represented a within-person approach to calculating MCID. In comparison, the exploratory static MCID estimate for the DRS correlated differences between GOSE and DRS scores across patients at a single time point. Because the MCID is typically used to determine a threshold of change that is clinically meaningful over time, the dynamic estimate was used as the primary analysis.

The dynamic MCID for the DRS was determined using all persons in the TBIMS NDB who experienced a change of both DRS and GOSE scores between year 1 and year 2 and had a GOSE score of 3–8 at year 1. This approach included persons in all three trend groups (*n* = 11,102) and specifically did not exclude persons in strong defiance of the trend (i.e., persons who had an improvement of GOSE with worsening or no change of DRS, or worsening of GOSE with improvement or no change of DRS; *n* = 2308). The dynamic approach therefore represented a real-world scenario, in which the MCID for the DRS was estimated to be a −0.68-point change, based on a 1-point change for the GOSE on a scale of −7 to 5 points that represented nearly the entire range of possible GOSE scores.

The subanalysis in which the dynamic MCID was determined for persons who followed the consistent trend or were in soft defiance of the trend (*n* = 8,794), but excluded persons in strong defiance of the trend estimated a higher MCID for the DRS of a −1.80-point change. The greater MCID estimate for the dynamic subanalysis was reflective of the elimination of persons who experienced worsening of DRS (*n* = 479) and persons who did not experience change of DRS (*n* = 1419), and likely represented measurement error.

The exploratory static MCID for the DRS was more conservative than the dynamic approach given that it only required persons to have a score for the DRS and GOSE at the 1-year time point, which may not have represented change over time. The static analysis estimated MCID using linear regression to determine the slope of the best-fit line, in this case over a GOSE score range of 3–8 points (i.e., low severe disability to upper good recovery), while eliminating persons who scored 1 (i.e., were dead) or 2 (i.e., were in a vegetative state) who accounted for 4.6% of persons. The slope of the best-fit line was −1.28, which indicated that a 1-point change of GOSE resulted in a −1.28-point change of DRS and thus the MCID for the DRS. The associated coefficient of determination for the linear regression was 0.49, meaning that 49% of the data points fell within the data line and that the DRS and GOSE were moderately correlated.

Alternative static analyses for GOSE score ranges of 3–7 and 3–6 were moderately correlated with the DRS and eliminated 20.7% of persons from the population who had a GOSE score of 8 and 33.6% of persons from the population who had GOSE scores of 7 and 8, respectively. The alternative static MCID estimates for the DRS in the GOSE ranges of 3–7 and 3–6 were −1.58 and −1.88 points, respectively. These findings demonstrated that the elimination of persons with the most favorable GOSE outcomes of 7 and 8 (i.e., low good recovery and upper good recovery) increased MCID estimates for the DRS, suggesting that persons with greater disability on the GOSE scale required a greater DRS score change to achieve MCID.

The final MCID for the DRS was determined using the triangulation method (i.e., calculation of the arithmetic mean), which resulted in a final MCID for the DRS of a −1.0-point change. Triangulation allowed the primary dynamic, within-person approach to have equal weight to the exploratory static approach that used scores at a single time point, the relative merits of which have been discussed previously. The triangulation method, which can be used with two or three components, allowed for a multi-layered approach to calculating MCID that is used widely across multiple therapeutic areas.^11,13,17–19^

The subanalysis, which triangulated the dynamic MCID of a −1.80-point change (consistent trend and in soft defiance of the trend) with the exploratory static MCID of a −1.28-point change, resulted in an MCID for the DRS of −1.5 points. This served as a sensitivity analysis, in which persons in strong defiance of the trend were excluded because they likely represented measurement error. In this study of a large population of persons with chronic TBI, the resulting higher MCID of −1.5 points for the DRS was consistent with a previous study by McCrea and colleagues, in which distribution- and anchor-based MCID estimates from a randomized controlled clinical trial of 61 persons with chronic TBI (mean DRS score, 4.6 points) were triangulated with a Delphi panel MCID estimate from a study by Mattke and colleagues.^12,13^ The Delphi panel MCID estimate for the DRS was defined by Mattke and colleagues as a −1-point change for persons with chronic TBI, based on the assessment of hypothetical vignettes by a multi-disciplinary expert Delphi panel.^12^ The vignettes focused exclusively on motor function, with persons having DRS scores of 2–5 points (i.e., persons had low disability), with MCID changes mostly observed in the DRS subscale domains of Level of Functioning and Employability.^12^

Distribution-based, anchor-based, and Delphi panel methods are alternatives to those used in this study to estimate the MCID. The distribution-based method references the MCID to a measure of variability or effect size in the scale of interest (e.g., 0.5 × SD of change), the anchor-based method references the MCID to an external measure of change (e.g., an alternative scale with an established clinically meaningful cutoff), and the Delphi panel that recruits a panel of experts to rate the MCID.^11,20,21^ The static method used in this study is similar to the anchor-based method, given that both approaches require a strong correlation to be established between the test scale and an established scale with a known MCID; the MCID of the established scale is then used to estimate the MCID of the test scale using a receiver operator characteristic curve.^11,13^

### Evaluating the clinical meaningfulness of a 1-point change across Disability Rating Scale subscale domains

As described previously, the DRS is a 30-point ordinal scale that assesses eight (A to H) functional areas, or subscale domains, which focus on neurological function and restrictions of daily living.^1,7^ In a study by Hammond and colleagues, which examined areas of change in the DRS from year 1 post-TBI to year 2 and year 5, most change over time was found to be in the DRS subscale domains of Level of Functioning (Area G) and Employability (Area H).^22^ A previous study by Malec and colleagues found that the addition of five items to the DRS subscale domains of Level of Functioning (Area G) and Employability (Area H), including a rating of a person's current employment, improved the sensitivity of the DRS to the milder levels of disability measured by the upper end of the scale.^16^

In this study, we defined the MCID for the DRS as a 1-point change, which was in common with a Delphi panel estimate made by Mattke and colleagues.^12^ However, the clinical meaningfulness of a 1-point change across the full range of the DRS is unclear. For example, in the DRS subscale domain of Eye Opening (Area A), a 1-point change can represent the difference between spontaneous eye opening and eye opening in response to speech or sensory stimulation,^23^ a difference that may or may not be clinically meaningful to the person. DRS subscale domains of Feeding, Toileting, and Grooming (Areas D to F) assess the person's cognitive ability to undertake these tasks, but do not assess their ability to carry them out.^23^ Further, a 1-point change in these functional areas may or may not be clinically meaningful. For example, in the DRS subscale domain of Feeding (Area D), a difference of 1 point can represent the difference between continuously showing awareness of how to feed and intermittently showing awareness of how to feed,^23^ which to the person may not be clinically meaningful.

In contrast, in the DRS subscale domains of Level of Functioning and Employability (Areas G and H),^23^ a 1-point difference clearly represents clinically meaningful change. For example, in the DRS subscale domain of Level of Functioning (Area G), a difference of 1 point can represent the difference between a person being completely independent, in which there are no restrictions of living, and being independent in a special environment, in which independence is limited by the requirement for mechanical aids.^23^ In the DRS subscale domain of Employability (Area H), a difference of 1 point can represent the difference between being able to compete for unrestricted employment and being competitive for selective employment because of psychosocial or physical limitations.^23^ Evaluating the full range of DRS in this study suggests that the MCID of a 1-point change has the most validity in the DRS subscale domains of Level of Functioning (Area G) and Employability (Area H).

### Study strengths and limitations

The strengths of this study included the: 1) use of the large, prospective, longitudinal TBIMS NDB, in which there were no notable differences between the study sample and the overall TBIMS NDB population, and 2) consistent correlations between the DRS and GOSE in the GOSE range of 3–8 points at years 1, 2, and 5 post-injury. The limitations of this study included: 1) the observational, uncontrolled design of the TBIMS NDB; 2) the retrospective nature of this analysis; and 3) that this study specifically included persons with moderate-to-severe TBI who received acute inpatient rehabilitation, and as such the findings may not necessarily translate to persons with mild TBI or those who do not receive acute inpatient rehabilitation.

## Conclusion

This retrospective analysis of up to 14,083 persons with chronic TBI from the TBIMS NDB correlated DRS change with the MCID for the GOSE of a 1-point improvement (GOSE score range, 3–8), determining the MCID for the DRS to be a −1.0-point change at year 1 follow-up and supporting the DRS as an alternative primary outcome measure for chronic TBI interventional studies, demonstrating how the findings of translational research may usefully support both clinical research and clinical management.

## Supplementary Material

Supplemental data

## Supplementary Material

Supplementary Figure S1A,B

## Abbreviations Used

CI: confidence interval
DRS: Disability Rating Scale
FDA: U.S. Food and Drug Administration
GCS: Glasgow Coma Scale
GOSE: Extended Glasgow Outcome Scale
MCID: minimally clinically important difference
NDB: TBIMS National Database
SD: standard deviation
TBI: traumatic brain injury
TBIMS: Traumatic Brain Injury Model Systems
