# Supplementary material for: Determining the Minimally Clinically Important Difference for the Disability Rating Scale in Persons With Chronic Traumatic Brain Injury
Source: Neurotrauma Rep. 2023 Jul 4;4(1):447–57. doi: 10.1089/neur.2023.0038 (PMC10354728; doi:10.1089/neur.2023.0038)
Supplement: Supplemental data [file Suppl_FigureS1.docx]

**FIGURE LEGEND**

**Supplementary Figure 1. Alternative Static Minimally Clinically Important Differences for DRS**

Alternative exploratory static MCIDs for DRS were determined by plotting DRS scores vs. GOSE score ranges of 3-7 and 3-6 points at 1-year post-injury, which resulted in slopes of the best fit lines and coefficients of determination (R^2^) of **(A)** -1.58 (R^2^: 0.46), and **(B)** -1.88 (R^2^: 0.44), respectively.
